# Secondary Reconstruction of Posttraumatic Enophthalmos with Titanium Mesh and Buccal Fat Pad Graft: Case Report

**DOI:** 10.1055/s-0037-1603982

**Published:** 2017-07-05

**Authors:** Gustavo Gaffrée, Roberto Santos, Viviane Cupello, José Emilio Polinati, Leonardo Paredes

**Affiliations:** 1 Department of Oral and Maxillofacial Surgery, Lourenço Jorge Hospital, Rio de Janeiro, Rio de Janeiro, Brazil

**Keywords:** enophthalmos, buccal fat pad graft, titanium mesh, blowout fractures

## Abstract

Secondary enophthalmos caused by an untreated orbital blowout fracture can cause esthetic and functional disturbances. The esthetic defect is manifested by sinking of the superior sulcus and the hypophthalmic globe. Functional impairment of the eye can usually be a common complaint with restriction of eye motion and diplopia. Early diagnosis followed by repair of surgically correctable fractures is the most acceptable procedure. Failure in the primary treatment may cause scar contraction and fat atrophy. The aim of this paper is to report a case of a late treatment of blowout orbital floor fracture with secondary enophthalmos using titanium mesh and buccal fat pad graft.

 One of the most frequent midfacial injuries is the orbital bone fracture. It corresponds to 40% of trauma injuries in the region. It is usually associated with other fractures such as those in the maxillomandibular, zygomatic, and frontal bones. Traumas that include the orbital bone and its adjoining soft tissue generally cause diplopia, ocular muscle entrapment, enophthalmos, and other serious subsequent events. Recent technologies in oculoplastic surgery and broad knowledge of the complex anatomy of the internal orbit that has emerged in the last 20 years have completely transformed the management of orbital trauma. The diagnosis and management of internal orbital fractures have greatly benefited from computed tomography (CT) scanning. Injuries to the internal orbit can now be determined and their size, location, and any displacement of bony walls and orbital soft tissues can be measured. Also, the overall severity can be assessed. Due to the extra data, we can not only observe but anticipate treatment to prevent the development of enophthalmos. 1 Late enophthalmos from unrepaired zygomatic or orbital blowout fractures can lead to functional and esthetic impairment. Sunken appearance of the superior sulcus and depressed globe are obvious cosmetic defects. Another aspect that can pose concerns is the functional impairment of the eye. The most frequent complaints are related to restriction of motion and diplopia. The most applicable procedure is the early repair of surgically correctable fractures as opposed to scar contraction or fat atrophy. 2 The objective of this paper was to present a case of late treatment of blowout orbital floor fracture with secondary enophthalmos. 

## Case Report

A 29-year-old female patient was referred by an ophthalmologist to the maxillofacial surgery unit of our hospital. She had a history of trauma to the right eye, which occurred 3 months before the appointment while she was practicing martial arts. She did not have any symptoms at the time and therefore decided not to seek specialist care. Her main complaint was progressive visual disturbance (binocular diplopia) associated with changes in the positioning of her right eye.

**Fig. 1:**
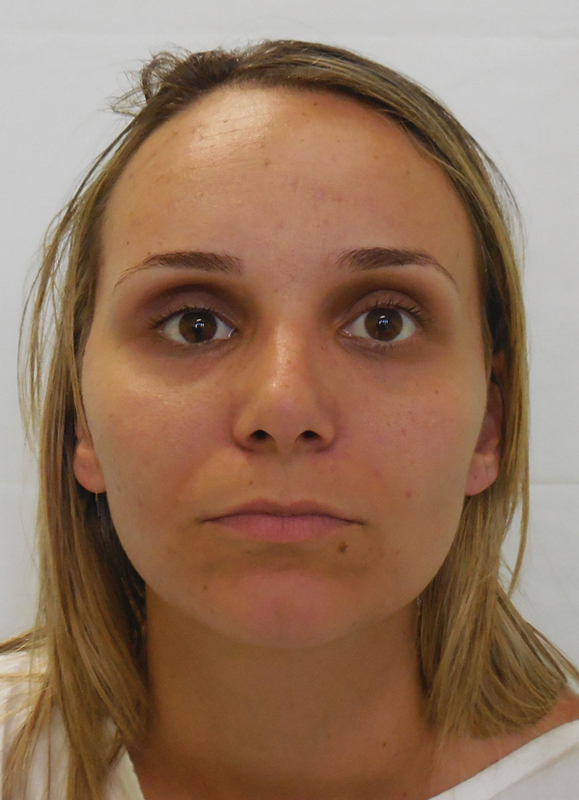
Patient presenting enophthalmos.

**Fig. 2:**
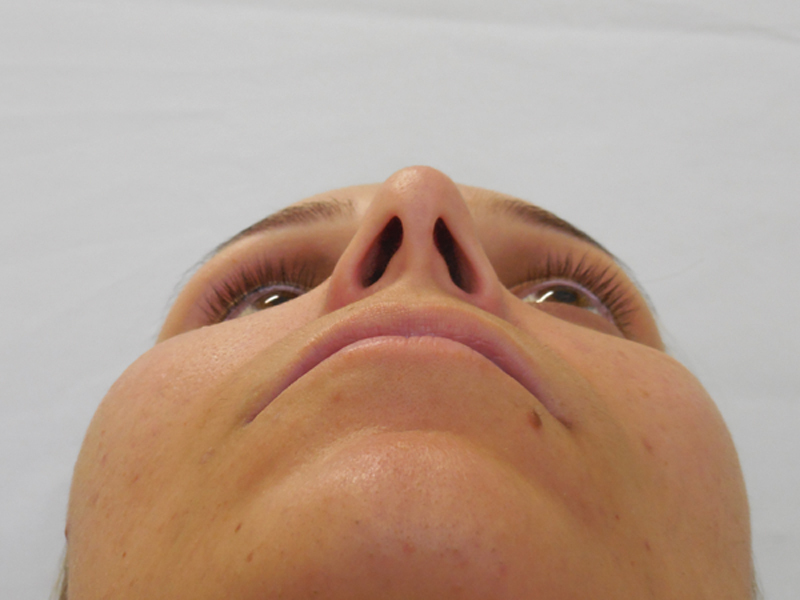
Axial view of enophthalmos.

 During the physical examination, we noted enophthalmos in the right eye with increase of superior eyelid groove and discreet unevenness in comparison to the left eye ( Figs. 1 and 2 ). Both pupillary reflex and extrinsic globe movement of the right eye were preserved. 

**Fig. 3:**
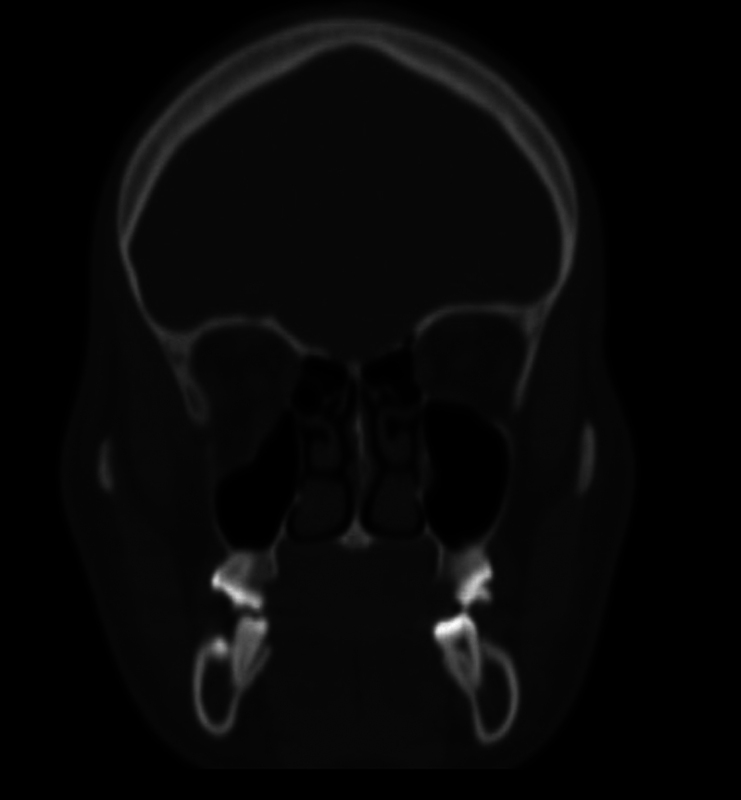
Computed tomographic (CT) scan of coronal posterior view.

**Fig. 4:**
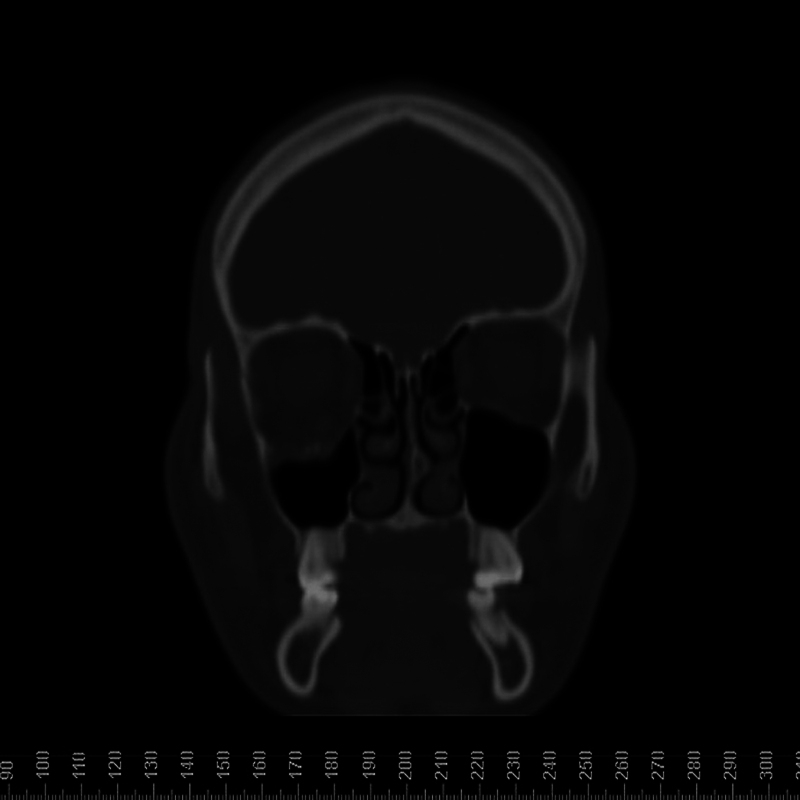
Computed tomographic (CT) scan of coronal anterior view.

 The CT scan revealed pure blowout fracture of the floor of the right orbit with huge fat herniation of the interior of the maxillary sinus, mainly in the posterior-lateral side of the orbit ( Figs. 3 and 4 ). 

**Fig. 5:**
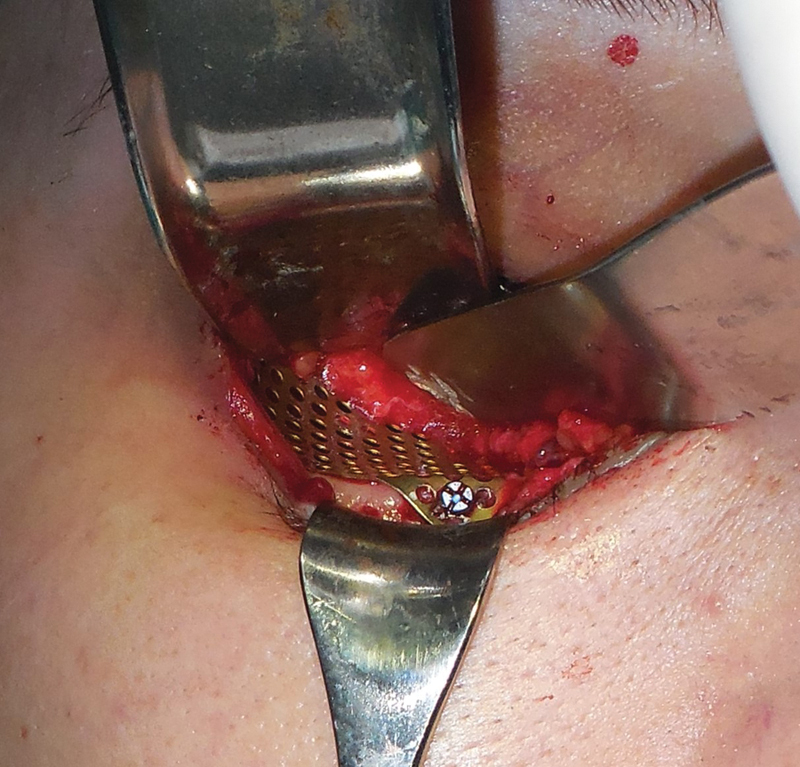
Reconstruction of titanium mesh.

**Fig. 6:**
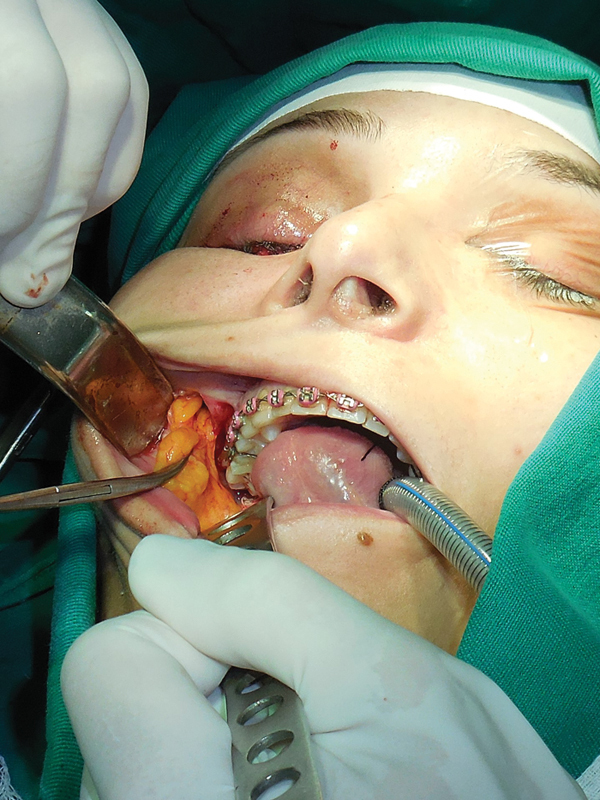
Harvesting the buccal fat pad.

**Fig. 7:**
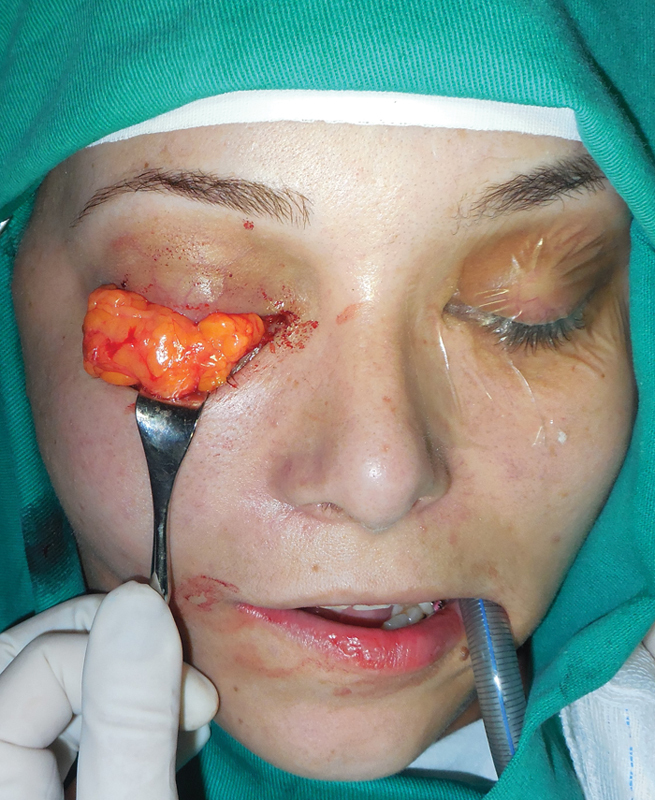
Buccal fat pad graft before placement into the orbital cavity.

**Fig. 8:**
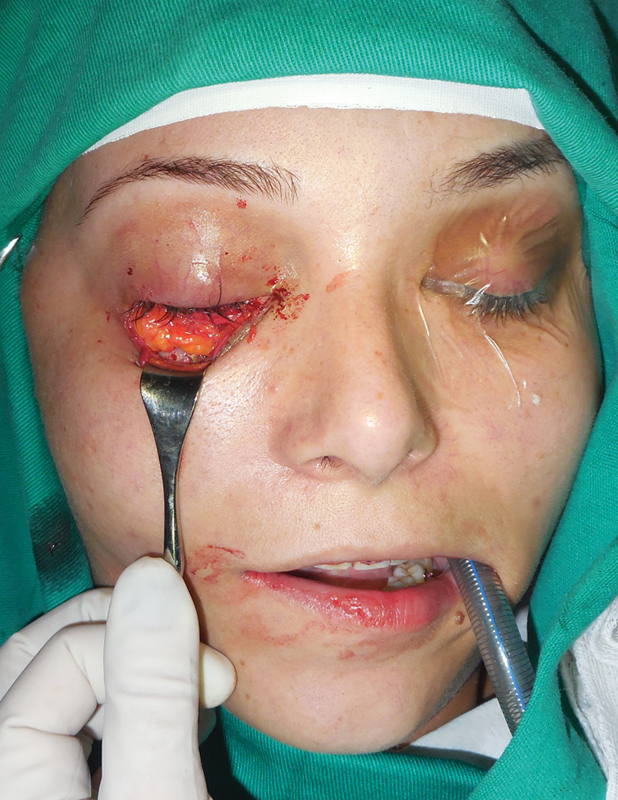
Buccal fat pad graft inserted into the orbital cavity.

 Surgery under general anesthesia was our choice. We performed a transconjunctival access with lateral canthotomy to reconstruct the orbital floor with 1.6-mm titanium mesh (OsteoMed, Addison, TX) fixated with two 1.6-mm screws ( Fig. 5 ). The buccal fat pad was harvested bilaterally with a small intraoral incision to prevent fat atrophy ( Fig. 6 ). The fat pad was inserted into the posterior portion of the orbital cavity, which allowed for an adequate projection of the globe ( Figs. 7 and 8 ). 

A forced duction test was performed and no restrictions in the eyeball movements were detected. Transconjunctival access was mainly closed with Vicryl 5.0 (Ethicon, USA)

Initial postsurgical care was uneventful. Visual acuity preservation was frequently monitored.

**Fig. 9:**
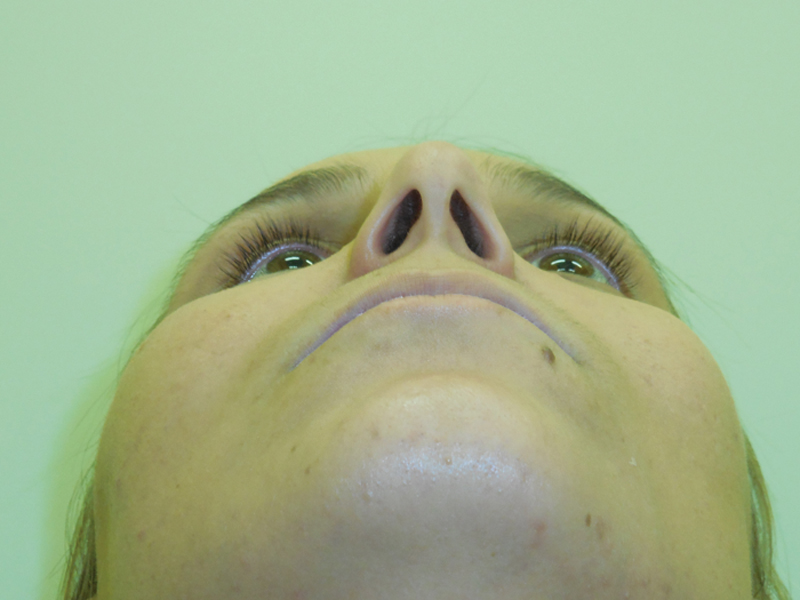
Postoperative axial view 4 months after surgery.

**Fig. 10:**
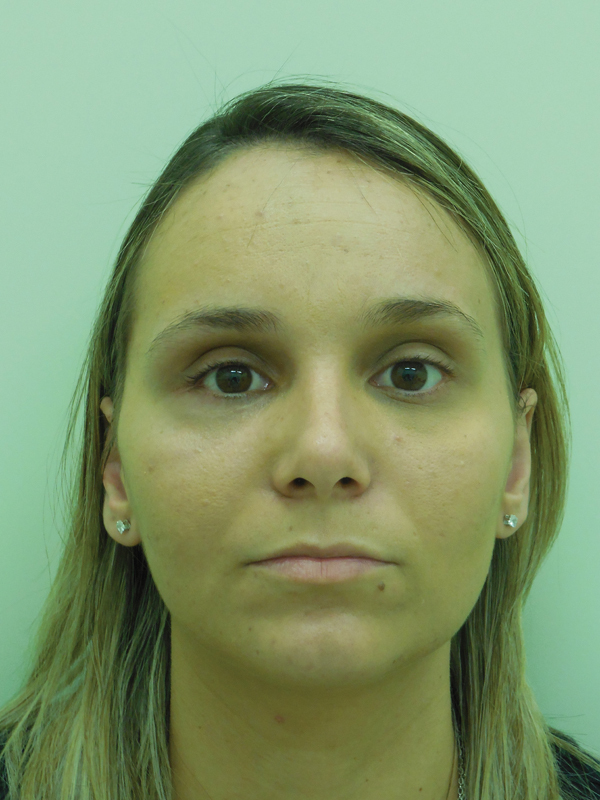
Four months postoperative view showing better position of globe.

 Four months past surgery, excellent eyeball projection, absence of diplopia, and preservation of extrinsic eyeball movements were observed ( Figs. 9 and 10 ). 

**Fig. 11:**
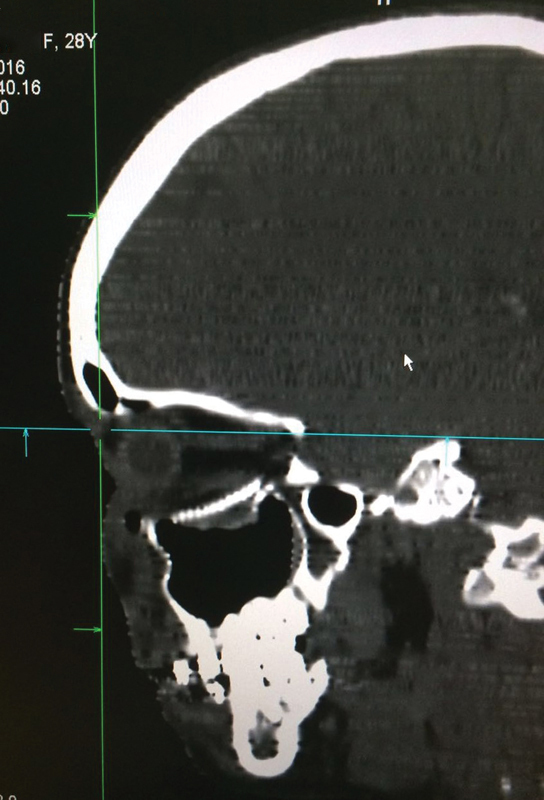
Computed tomographic (CT) scan 4 months postoperatively showing adequate reconstruction of floor of orbit.

**Fig. 12:**
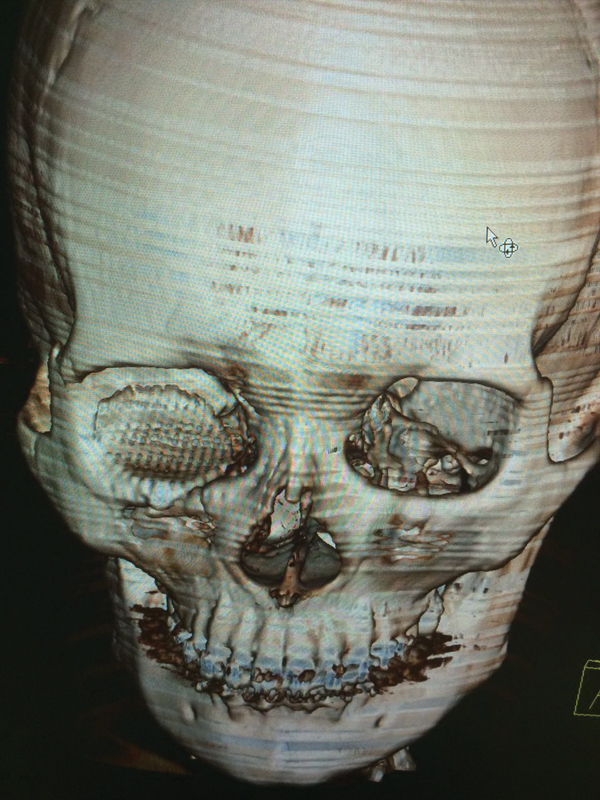
Three-dimensional computed tomographic (3D CT) scan 4 months postoperatively showing reconstruction of orbital floor.

 Good reconstruction of the fractured boundary of orbital walls was clear in the control CT scan ( Figs. 11 and 12 ). 

## Discussion

 Enophthalmos is characterized by a posterior displacement of the ocular globe within the bony orbit. Enophthalmos of the size 2 to 3 mm can be detected clinically. An enophthalmos of more than 5 mm is disfiguring. Enophthalmos is characterized by a displacement of a relatively constant volume of orbital soft tissue into an enlarged bony orbit. Key factors in this incongruity of soft tissue and orbital volume are scar contractures and fat atrophy. Since it is frequently present in patients with inadequately treated orbital fractures with disruption of orbital floor, enophthalmos is usually accompanied by inferior displacement of the globe. This recession of the globe changes the drape of the upper lid on the globe and tends to deepen the superior tarsal fold, which causes ptosis of the upper lid. The range of reported incidence of enophthalmos associated with facial bone fractures varies, from 12.5 to 65% in patients who suffered high-energy trauma. 3

 Several authors believe that to repair orbital fractures early, that is, before edema or after its resolution is the best option to facilitate appropriate surgical reduction and fixation. Immediate surgical intervention for orbital fractures is seldom recommended in “trapdoor fracture,” which is a significant enophthalmos in association with orbital soft-tissue entrapment that causes oculocardiac reflex and diplopia. 4 5 When there is no urgent indication of surgery for repair of the orbital floor, a 2-week period of observation is recommended for most orbital floor fractures. 6 Still, an extended time for observation prior to surgical intervention may result in suboptimal outcomes. It was reported that delays longer than 2 months in the reconstruction of the orbital floor led to inferior outcomes when compared with early surgery. 6 Dulley and Fells 7 noted that 20% of the patients who had surgery within 2 weeks of trauma had enophthalmos while for patients who underwent surgery 6 months after the initial trauma, the percentage was 72. The patient in this case underwent surgery 2 months after the trauma with no previous treatment. 

 The approach to the site of the fracture depends on the type of injury, experience of the surgeon, and on the available equipment. Subciliary, subtarsal, and transconjunctival incisions are the most frequently used approaches. Subciliary approaches have been associated with much higher complication rates and 12.9% of cases result in ectropion. 8 Subtarsal approach is associated with less ectropion and depending on its correct placement, conspicuous scar rates are low, around 1 to 3%. 9 10 11 The preference for most surgeons is a transconjunctival approach of the orbital floor due to its very low complication rates (less than 1% in several series) and absence of a visible scar. 12 13 14

It is indisputable that restoration of the pretrauma volume of the internal orbit is the most critical component of orbital reconstruction. Reconstruction of the internal orbit is performed to restore its preinjury anatomy with an expected result of accurate globe position. In the process of reconstructing injuries in the patient's orbital wall, the surgeon merely determines the defect through the identification of bone edges and the spanning of the defect through an implant or autogenous graft.

 Titanium meshes are highly biocompatible, that is, they simply adjust to architecturally fit simple and complex defects, providing a robust support and not altering the shape or location over time. They can be adequately fixed to adjacent bone. Its osseointegration is well recognized; it can be sterilized easily and made readily available even though it may pose a higher cost. Unfortunately, the holes in the plates allow tissue ingrowth that may make removal more difficult. Also, cut edges are prone to snaring periorbital soft tissue during placement. The usefulness of titanium mesh implants was shown in a previous study comparing titanium meshes and calvarial bone grafts. Statistically significant improvement was found in the reconstructed orbital volume in cases where more than one orbital wall was fractured. This improvement was shown through measurements of orbital volume pre- and postreconstruction in patients reconstructed with titanium mesh. 1

 The utilization of CT-based mirroring-reconstruction images of the orbit was demonstrated in 22 patients with late posttraumatic enophthalmos. The authors manufactured anatomically adaptive titanium meshes through computer-aided design (CAD) and computer-aided manufacturing (CAM) techniques. The use of this implant reduced the trauma-induced increment in orbital volume by 65% and was able to correct 50% of severe late enophthalmos. Nevertheless, additional augmentation of orbital contents was necessary for further correction. 15 Nkenke et al 16 confirmed that both individually prefabricated CAD/CAM glass-bioceramic implants and nonpreformed titanium meshes had similar outcomes in the correction of secondary enophthalmos. 

 Even though the accepted pathogenesis for late enophthalmos is the enlargement of the bony orbit, many authors have lately tried to prevent or correct this deformity through augmentation of periorbital soft tissue. Numerous studies have shown that the degree of enophthalmos is concurrent with the loss of intraconal fat. The fat volume in CT sections was calculated and accentuated a 5% retrobulbar fat reduction in the enophthalmic cases. 3 Ramieri et al 17 affirmed that posterior fat was reduced and fragmented by scarring tissue. Ilankovan et al 18 declared that as retrobulbar fat accounts for the majority of the orbital volume (∼70%), fat atrophy and necrosis play a key role in the development of enophthalmos. 

 The utilization of fat grafts in the intraconal space was described in the treatment of enophthalmic orbit by use of a sharp needle. 5 The effects of intraconal fat grafting in patients with posttraumatic enophthalmos was researched by Hunter and Baker; 19 the procedure was performed in patients with healthy eyeballs or in those with anophthalmic sockets. The research proved that enophthalmos is stabilized within 3 months with this technique. The overall results were good but in 64% of cases, it was necessary to apply multiple injections. Malet 20 described a reliable number of anophthalmic socket patients who were treated with injections into the deep upper eyelid sulci. Hardy et al 21 presented the details of a retrospective study that enrolled 12 patients with anophthalmic and enophthalmic orbital cavity. In many studies, abdominal fat was used to graft the internal orbit. It was Bichat who in 1802 first defined the buccal fat pad as fat tissue. It is in the masticatory space and comprises a central body (corpus) with four extensions, namely buccal, pterygoid, superficial, and deep temporal. Fifty percent of the buccal fat pad is made up of the body and buccal extension. These are the portions of the buccal fat pad that can be used as donor sites for fat tissue grafts and can be reached through the oral cavity. In this case, we used a buccal fat pad graft since it can be harvested in a simple form and has a low morbidity rate. In this patient, we harvested the buccal fat pad bilaterally to prevent postoperative facial asymmetry. 

## Conclusion

Reconstruction of alloplastic orbital floor associated with endo-orbital fat grafting proves to be an optimal method to achieve proper globe support and positioning even in cases of correction of secondary enophthalmos. Reconstruction of alloplastic orbital floor associated with endo-orbital fat grafting has demonstrated to be an alternative method to achieve proper globe support and positioning even in cases of correction of secondary enophthalmos. Nonetheless, more research is necessary to assess the benefits of this choice of treatment.
